# Left Ventricular Mass in Dialysis Patients, Determinants and Relation with Outcome. Results from the COnvective TRansport STudy (CONTRAST)

**DOI:** 10.1371/journal.pone.0084587

**Published:** 2014-02-05

**Authors:** Ira M. Mostovaya, Michiel L. Bots, Marinus A. van den Dorpel, Roel Goldschmeding, Claire H. den Hoedt, Otto Kamp, Renée Levesque, Albert H. A. Mazairac, E. Lars Penne, Dorine W. Swinkels, Neelke C. van der Weerd, Piet M. ter Wee, Menso J. Nubé, Peter J. Blankestijn, Muriel P. C. Grooteman

**Affiliations:** 1 Department of Nephrology, University Medical Center Utrecht, Utrecht, the Netherlands; 2 Julius Center for Health Sciences and Primary Care, University Medical Center Utrecht, Utrecht, the Netherlands; 3 Department of Internal Medicine, Maasstad Hospital, Rotterdam, the Netherlands; 4 Department of Pathology, University Medical Center Utrecht, Utrecht, the Netherlands; 5 Department of Cardiology, Vrije Universiteit Medical Center, Amsterdam, the Netherlands; 6 Department of Nephrology, Centre Hospitalier de l'Université de Montréal St. Luc Hospital, Montréal, Canada; 7 Department of Nephrology, Vrije Universiteit Medical Center, Amsterdam, the Netherlands; 8 Department of Laboratory Medicine, Laboratory of Genetic, Endocrine and Metabolic diseases, Radboud University Medical Centre Nijmegen, the Netherlands; 9 Institute for Cardiovascular Research Vrije Universiteit Medical Center, Vrije Universiteit Medical Center, Amsterdam, the Netherlands; University of Florida, United States of America

## Abstract

**Background and Objectives:**

Left ventricular mass (LVM) is known to be related to overall and cardiovascular mortality in end stage kidney disease (ESKD) patients. The aims of the present study are 1) to determine whether LVM is associated with mortality and various cardiovascular events and 2) to identify determinants of LVM including biomarkers of inflammation and fibrosis.

**Design, Setting, Participants, & Measurements:**

Analysis was performed with data of 327 ESKD patients, a subset from the CONvective TRAnsport STudy (CONTRAST). Echocardiography was performed at baseline. Cox regression analysis was used to assess the relation of LVM tertiles with clinical events. Multivariable linear regression models were used to identify factors associated with LVM.

**Results:**

Median age was 65 (IQR: 54–73) years, 203 (61%) were male and median LVM was 227 (IQR: 183–279) grams. The risk of all-cause mortality (hazard ratio (HR) = 1.73, 95% CI: 1.11–2.99), cardiovascular death (HR = 3.66, 95% CI: 1.35–10.05) and sudden death (HR = 13.06; 95% CI: 6.60–107) was increased in the highest tertile (>260grams) of LVM. In the multivariable analysis positive relations with LVM were found for male gender (B = 38.8±10.3), residual renal function (B = 17.9±8.0), phosphate binder therapy (B = 16.9±8.5), and an inverse relation for a previous kidney transplantation (B = −41.1±7.6) and albumin (B = −2.9±1.1). Interleukin-6 (Il-6), high-sensitivity C-reactive protein (hsCRP), hepcidin-25 and connective tissue growth factor (CTGF) were not related to LVM.

**Conclusion:**

We confirm the relation between a high LVM and outcome and expand the evidence for increased risk of sudden death. No relationship was found between LVM and markers of inflammation and fibrosis.

**Trial Registration:**

Controlled-Trials.com ISRCTN38365125

## Introduction

Increased left ventricular mass (LVM) has been well described as a frequent component of end stage kidney disease (ESKD) [1]. In fact, more than seventy percent of patients starting dialysis show left ventricular hypertrophy (LVH) on echocardiography [2]. An increase in left ventricular mass (LVM) is associated with cardiovascular morbidity and mortality [3], [4]. Although the relation between LVM and overall mortality and cardiovascular events has been well established in ESKD patients, the association between LVM and certain types of cardiovascular morbidity (such as coronary heart disease: CHD) and mortality (such as sudden death) has not yet been thoroughly investigated.

Several inflammatory biomarkers associated with cardiovascular pathology and morbidity have been described for patients with chronic kidney disease (CKD). High sensitivity C-reactive protein (hsCRP and interleukin-6 (Il-6) are both well accepted markers of inflammation, related to increased risk of death and cardiovascular disease [5]. HsCRP is an acute phase reactant, which has been associated with an increased risk of major cardiovascular disease [6]. HsCRP levels are higher in HD patients than in healthy individuals [7] and have been shown to be independent predictors of LVM indexed for body surface area (LVMi) in CKD patients [8]. Il-6 is a short acting protein secreted by cells of the immune system in response to inflammatory stimuli, and is suspected to be a central regulator in the inflammatory process that leads to atherosclerosis [9]. Several studies have reported the relation between a high Il-6 and increased risk of developing CVD [10]–[12]. In patient deceased from acute myocardial infarction, Il-6 has been associated with mechanisms of cardiac hypertrophy [13]. Furthermore, Il-6 levels are increased in dialysis patients [7], [14].

Connective tissue growth factor (CTGF) is a signalling protein involved in the pathogenesis of renal and cardiac fibrosis [15]. In animal studies CTGF has been described to contribute to development of cardiac hypertrophy [16], [17]. CKD patients have a higher plasma CTGF level then healthy individuals, since CTGF is eliminated predominantly by the kidney [18].

Hepcidin-25 is a peptide produced by the liver, which regulates intestinal absorption of iron and its distribution through the body [19]. The gene encoding for hepcidin-25 is regulated in response to anemia, hypoxia and inflammation [20]. Furthermore, hepcidin-25 is related to increased risk of cardiovascular events in chronic hemodialysis patients [21].

Although several studies have described a relationship between hsCRP and left ventricle geometry and function [8], [22], [23], the relationship between LVM and the four described biomarkers has not been examined in a large population of HD patients.

We hypothesize that a high LVM will be related to a higher risk of mortality and cardiovascular events in our study, as is the case in previously studied dialysis populations. Furthermore we expect to find a positive relation between specific cardiovascular events such as risk of CHD or sudden death and LVM. Regarding hsCRP, Il-6, CTGF and hepcidin-25, since these markers are related to pathophysiological mechanisms that could theoretically promote increase of LVM, we assume to find a positive relation between the magnitude of LVM and hsCRP, Il-6, CTGF and hepcidin-25. Hence, the aims of this study are 1) to determine whether LVM is associated with mortality and various cardiovascular events in our population of ESKD patients and 2) to identify determinants of LVM including biomarkers of inflammation, systemic iron homeostasis and fibrosis in HD patients.

## Materials and Methods

### Patients

The present study included a subset of patients participating in the CONvective TRAnsport STudy (CONTRAST): 327 hemodialysis patients from 15 dialysis centres (14 Dutch centers and 1 Canadian center). CONTRAST has been designed to investigate the effects of increased convective transport by online HDF as compared with low-flux HD on all-cause mortality and cardiovascular morbidity and mortality (ISRCTN38365125) and included a total of 714 patients [24].

The study was conducted in accordance with the Declaration of Helsinki and approved by the medical ethics review boards of all participating dialysis centres. Written informed consent was obtained from all patients prior to enrolment. The names of the medical ethics committees/review boards that have approved this study are listed in the appendix S1 in File S1.

### Data collection

Baseline patient and dialysis characteristics were used for this analysis: information on demography, anthropometrics, medical history, medication and standard laboratory values. A history of cardiovascular disease was defined as a previous acute myocardial infarction, coronary artery bypass graft, percutaneous transluminal coronary angioplasty, angina pectoris, stroke, transient ischemic attack, intermittent claudication, amputation, percutaneous transluminal angioplasty, peripheral bypass surgery and renal percutaneous transluminal angioplasty.

Systolic and diastolic blood pressure was measured before and after three consecutive dialysis sessions at baseline using a standard electronic sphygmomanometer. The average of these measurements was computed and used for analysis.

The primary outcome of CONTRAST was all cause mortality. Cause of death was recorded and subdivided into cardiovascular mortality (fatal myocardial infraction, fatal cerebrovascular accident, fatal decompensatio cordis, a rupture of the abdominal aorta or sudden death) and non-cardiovascular mortality. Sudden death was defined as death within 1 hour of the onset of symptoms as verified by a witness.

The main secondary endpoint was a composite of fatal and non-fatal cardiovascular events. Cardiovascular events were defined as death from cardiovascular causes, non-fatal myocardial infarction, non-fatal stroke, therapeutic coronary procedure (percutaneous transluminal coronary angioplasty and/or stenting), therapeutic carotid procedure (endartrectomy and/or stenting), and vascular intervention not related to vascular access (revascularisation, percutaneous transluminal angioplasty and/or stenting) or amputation. Congestive heart failure was excluded as a cardiovascular event, since the distinction with fluid overload is often difficult to make in patients with end stage renal disease.

Follow-up of patients with respect to mortality and non-fatal cardiovascular events was continued even after they stopped with the randomized treatment because of a renal transplant (n = 71), a switch to peritoneal dialysis (n = 5), a move to another non-CONTRAST hospital (n = 11) or a stop of participation for other reasons (n = 58).

An independent Endpoint Adjudication Committee reviewed source documentation for all primary outcome events (deaths), as well as non-fatal cardiovascular events and infections.

### Laboratory measurements

Standard laboratory samples were analysed in the local laboratories of the participating hospitals by standard laboratory techniques.

Furthermore, in centres where storage of blood samples was logistically feasible, additional blood samples were drawn for the analysis of hsCRP, Il-6, CTGF and hepcidin prior to dialysis. Samples were placed on ice, and centrifuged within 30 min, at 1500 g for 10 minutes, and were stored at −80°C until assayed. A total of 248 patients, out of the 327 who underwent echocardiography, were treated in such centers and therefore had additional measurements of hsCRP, Il-6, CTGF and hepcidin.

High sensitivity CRP, hepcidin-25, CTGF and IL-6 levels were measured centrally. Measurements of the bioactive hepcidin-25 were performed with time of flight mass spectrometry which has been described previously [25]. High sensitivity CRP (mg/L) was measured with a particle-enhanced immunoturbidimetric assay on a Roche-Hitachi analyzer as described elsewhere [21]. IL-6 (pg/mL) was measured with an ELISA (Sanquin, Amsterdam, The Netherlands), details have been described earlier [26]. CTGF levels in plasma were determined by sandwich ELISA, using two specific antibodies (FibroGen Inc., San Francisco, CA, USA) directed against two distinct isotopes in the amino-terminal fragment of CTGF, detecting both full length CTGF and the N-fragment, as shown earlier [18].

### Echocardiographic measurements

In 15 centres, patients were requested to undergo 2-dimensional echocardiography next to the standard CONTRAST baseline data collection.

Transthoracic echocardiography studies were performed on a mid-week non-dialysis day by an echocardiographer at the participating local hospital. From the parasternal long axis position the left ventricular end-diastolic diameter (LVEDD), end-systolic diameter (LVESD) as well as the posterior and septal wall thickness were determined. The ultrasound investigations were then assessed by an independent experienced echocardiographer at the core laboratory (VU medical Center, Amsterdam, the Netherlands), who was blinded for other patient data. LVM was calculated using the formula of Devereux and Reickek [27], modified in accordance with the recommendations of the American Society of Echocardiography [28]. LVH was defined as an LVM/height^2.7^ >44g/m^2.7^ for women and >48 g/m^2.7^ for men [3].

### Data analysis

Data were reported as proportions or as means with standard deviation (SD) or medians with inter-quartile ranges (IQR) when appropriate.

The average percentage of missing values per variable was 7.7%. No data were missing regarding clinical events. Multiple imputation was performed on all variables, where <40% of data were missing. One variable was not imputed due to a higher percentage of missing values, namely blood flow. Imputation was performed to prevent bias in reported estimates and to improve statistical power [29].

To study the independent relation of each variable with LVM, linear regression analysis was used. Patient and dialysis related variables that showed a univariable relation with LVM using a cut-off p-value <0,20 were entered in a multivariate model in consequent groups: demographic data, patient history, dialysis properties, therapeutic parameters and haemodynamic measurements. In addition, height and weight were added into the model upfront.

In a separate analysis, the variables hsCRP, Il-6, hepcidin-25 and CTGF were added to the constructed multivariate model one at a time. The old and new models were compared based on direction of the estimate and the significance of the regression coefficient of the added marker.

The relations between LVM and all-cause mortality, as well as cardiovascular events, cardiovascular death, sudden death and CHD were evaluated by Cox proportional hazards models, involving the time to the first relevant endpoint in any individual patient. For this analysis LVM was both analysed as a linear variable and divided into categories (tertiles). The number of events (in particular sudden death and CHD events) was small, and thus adjusting for all relevant possible confounders would lead to an overfitted model. Propensity scores as opposed to individual variables were used to adjust the models thus omitting the problem of an overfitted model. The propensity score [30] model estimated each individuals probability of having an LVM above the median of the studied population. Propensity score was built using a logistic model including all variables associated with LVM with p<0.20. Moreover, height, post-dialysis baseline weight and dialysis modality (intervention) were added into the propensity score model upfront.

Results were considered statistically significant when p<0.05 (two-sided). All calculations were made by use of a standard statistical package (SPSS for Windows Version 18.0.1; SPSS Inc. Headquarters, Chicago, Illinois, US).

## Results

327 patients participating in CONTRAST underwent echocardiography. Out of this group, in 248 patients blood was collected for a measurement of markers of inflammation and fibrosis. Median age was 65 (IQR: 54–73) years, 203 were male (61%) and the median dialysis vintage was 2.0 (IQR: 1.0–4.0) years. Median LVM was 227 (IQR: 183–279) grams. A total of 230 patients (71%) had LVH. The baseline characteristics of the whole CONTRAST cohort and of the echocardiography population are shown in table 1. The mean follow-up time was 2.0 (minimum 0.1, maximum 6.5) years. Within the group of patients with an LVM measurement 130 (39.8%) patients died from any cause and 116 (35.5%) had a cardiovascular event, out of which 43 (13.1%) were fatal. CHD (angina pectoris or acute myocardial infarction) occurred in 53 (16.2%) patients, of whom 3 (0.9%) died. Sudden death occurred in 24 (7.3%) patients.

**Table 1:** Demographic, anthropometric, biochemical, hemodynamic and dialysis characteristics of the study population.

	Total Cohort	Echo cor cohort
	n = 714	n = 327
***Demographic data***		
Male gender	445 (62%)	200 (61%)
Race, Caucasian	304 (85%)	263 (80%)
Age, years	64.1±13.7	63.0±13.3
Smoking	133 (19%)	66 (20%)
***Anthropometrics***		
Length (cm)	168±10	168±11
Weight (kg)	72.4±14.4	72.1±14.3
BMI (kg/m^2^)	25.4±14.4	25.5±4.9
Body Surface Area (m^2^)	1.85 (0.28)*	1.85 (0.30)*
***Dialysis Properties***		
Dialysis vintage (years)	1.8 (1.0–4.0)*	2.0 (1.0–4.0)*
Duration of dialysis (minutes)	226±23	225±23
Blood flow (mL/minute)	300 (300–348)*	300 (300–350)*
spKt/Vurea	1.40±0.22	1.39±0.20
AV fistula	279 (78%)	260 (80%)
Patients with residual kidney function	186 (52%)	171 (52%)
***Comorbidities***		
Cardiovascular disease	313 (44%)	146 (45%)
Diabetes	170 (24%)	83 (25%)
Previous kidney transplant	78 (11%)	30 (9%)
***Laboratory parameters***		
Hemoglobin (g/dL)	11.8±0.40	11.8±1.3
Phosphate (mmol/L)	1.64±0.49	1.67±0.50
Calcium (mmol/L)	2.31±0.18	2.30±0.18
Albumin (g/L)	40.4±3.8	41.2 (37.9–43.5)*
Creatinine (µmol/L), pre-dialysis	861±255	883±252
hsCRP (mg/L)	-	4.0 (1.6–11.9)*
Il-6 (pg/mL)	-	2.0 (1.2–3.8)*
CTGF (nmol/L)	-	3.6 (2.8–4.3)*
Hepcidin -25 (nM)	-	14.2 (6.3–22.4)*
Ferritin (ng/mL)	-	377 (211–597)*
TSAT (%)	-	22 (15–29)*
***Medication***		
Erythropietin therapy	314 (88%)	295 (91%)
Diuretic therapy	250 (35%)	129 (39%)
Beta-blocker therapy	184 (51%)	174 (53%)
RAS inhibitor therapy	179 (50%)	162 (50%)
Lipid lowering therapy	196 (55%)	152 (47%)
Vitamin D administration	227 (63%)	222 (68%)
Phosphate binding therapy	445 (62%)	194 (59%)
Platelet aggregation therapy or coumarines	111 (34%)	122 (36%)
Iron supplements	476 (67%)	213 (65%)
***Hemodynamic measurements***		
Systolic blood pressure (mm Hg)	147±21	142±19
Diastolic blood pressure (mm Hg)	75±12	74±10
LVEDD (mm)	-	10 (9–11)*
LVESD (mm)	-	32 (27–38)*
EFLV (%)	-	65 (55–72)*
LVM (g)	-	227 (183–279)*
LVH	-	230 (71%)

* :median and IQR (P25–P75).

AV: arterio-venous;BMI: mody mass index; CTGF: connective tissue growth factor; EFLV: ejection fraction of left ventricle; hsCRP: high sensitivity C-reactive protein; Il-6: interleukin 6; LVEDD: left ventricular end diastolic diameter; LVESD: left ventricular end systolic diameter; LVH: left ventricular hypertrophy; LVM: left ventricular mass; RAS: renin-angiotensin system; TSAT: transferrin saturation.

### Relation to LVM and outcome

**Figure 1:**
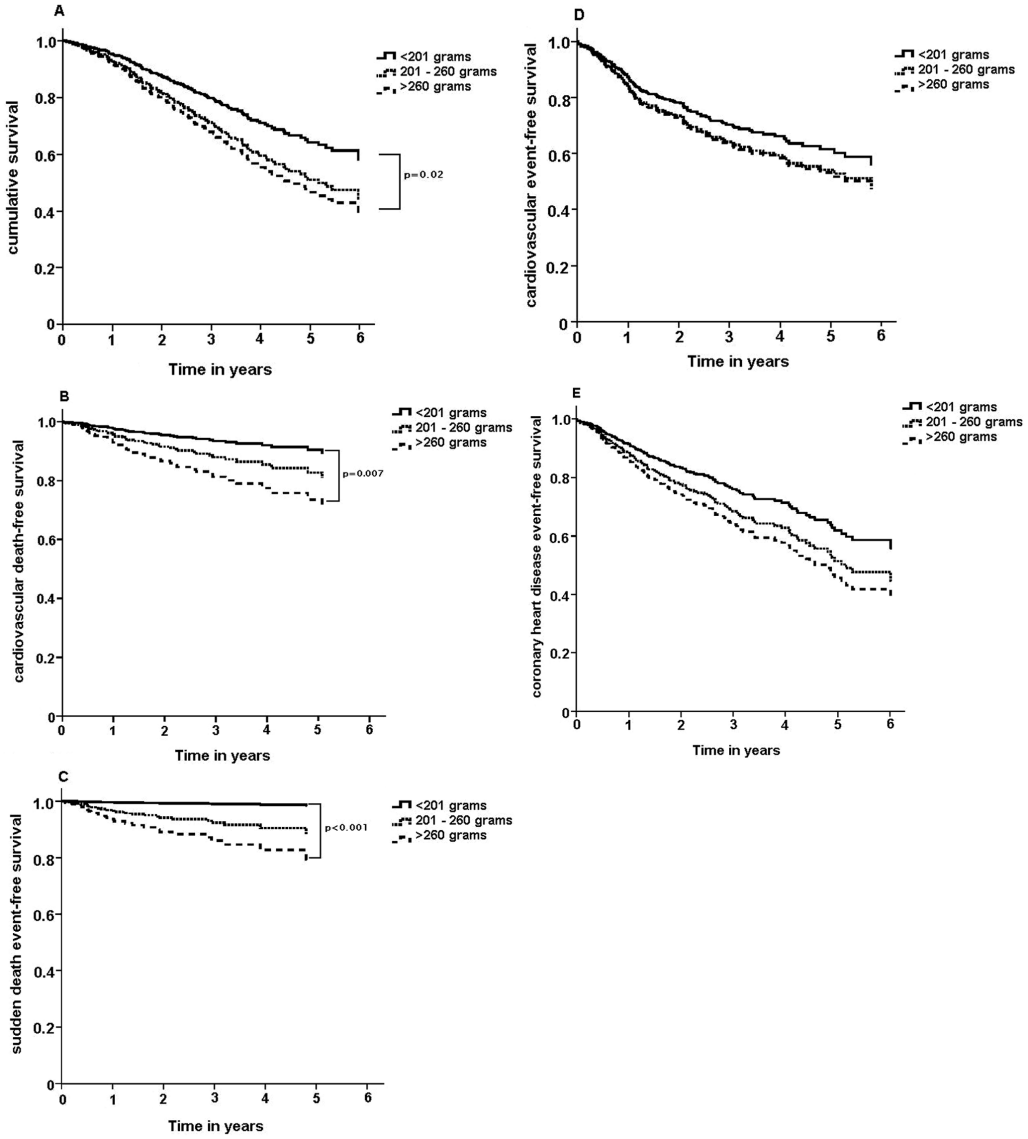
Survival curves for (A) time to death from any cause, (B) cardiovascular death, (C) sudden death, (D) cardiovascular events (both fatal and non-fatal), (E) coronary heart disease events (both fatal and non-fatal, all stratified by LVM tertiles and adjusted using propensity scores.

Table 2 shows proportional hazard ratios for all-cause mortality, cardiovascular death, sudden death, combined fatal and non-fatal cardiovascular events and CHD events; both crude and adjusted using propensity scores. Risk of all-cause mortality, cardiovascular death and sudden death was increased in the highest tertile (>260grams) of LVM; while no difference in risk was found for overall cardiovascular events and CHD events in the LVM tertiles. Figure 1 shows survival curves for the clinical events described above stratified by LVM tertiles.

**Table 2:** Hazard ratio of clinical events by LVM in grams divided into tertiles.

	T1: <201	T2: 201<LVM<260	95% CI	T3: >260	95% CI
**Crude**					
Mortality	1	1.61*	1.01–2.55	2.17*	1.39–3.38
Cardiovascular death	1	2.24	0.90–5.55	3.76*	1.61–8.82
Sudden death	1	8.93*	1.12–71.4	17.8*	2.35–135.0
Cardiovascular events	1	1.47	0.92–2.44	1.66*	1.06–2.67
CHD events	1	1.04	0.51–2.13	1.13	0.56–2.31
**Adjusted** a					
Mortality	1	1.50	0.92–2.10	1.73*	1.11–2.99
Cardiovascular death	1	1.80	0.64–5.07	3.69*	1.35–10.05
Sudden death	1	6.29	0.72–52.70	13.06*	6.60–107.16
Cardiovascular events	1	1.27	0.74–2.18	1.49	0.85–2.60
CHD events	1	1.22	0.71–2.09	1.51	0.87–2.64

* p<0.05.

a Adjusted with a propensity score containing determinants of LVM (male gender, residual renal function, history of kidney transplantation, albumin, use of RAS-inhibitors, use of phosphate binders, systolic blood pressure) and history of cardiovascular disease, diabetes, height, post-dialysis weight and dialysis modality (intervention).

As shown in Table S1a and S1b in File S1, when LVM was indexed for BSA or height^2.7^, relations with clinical events were similar.

### Determinants of LVM

The univariable and multivariable analysis results of LVM are shown in Table 3. In the multivariate analysis significant positive relations with LVM were found for male gender, presence of residual renal function and phosphate binder therapy. There were inverse relations for a history of kidney transplantation and albumin. The complete-case multivariate regression analysis showed similar results as demonstrated in Table S2 in File S1.

**Table 3:** Determinants of LVM in dialysis patients: univariable and multivariable regression analysis.

	Univariable model		Multivariable model	
Determinant	B	95% CI	B	95% CI
***Demographic data***				
Male gender	56.47	39.03 to 73.90	38.80	18.64 to 58.96
Race, Caucasian	12.92	−9.75 to 35.60		
Age (years)	0.75	0.08 to 1.42		
Smoking	22.20	−0.47 to 44.87		
***Dialysis Properties***				
Duration of dialysis (hours)	35.14	10.95 to 59.33		
spKt/Vurea	−102.7	−145.7 to −59.75		
AV fistula	17.59	−4.66 to 38.83		
***Comorbidities***				
Cardiovascular disease	16.54	−1.50 to 34.58		
Diabetes	1.94	−18.45 to 22.37		
Previous kidney transplant	−49.76	−80.38 to −19.01	−41.12	−55.94 to −26.31
Dialysis vintage (years)	−5.45	−8.61 to −2.30		
Residual kidney function	29.28	−11.52 to 47.04	17.88	2.16 to 33.61
***Laboratory parameters***				
Hemoglobin (g/dL)	−1.00	−12.53 to 10.53		
Phosphate (mmol/L)	0.89	−17.17 to 18.94		
Calcium (mmol/L)	13.37	−32.36 to 63.99		
Calcium*Phosphate	1.28	−6.48 to 9.03		
Albumin (g/L)	−1.99	−4.17 to 0.20	−2.94	−5.08 to −0.81
Creatinin (µmol/L)	−0.02	−0.05 to 0.02		
***Therapeutic parameters***				
Erythropietin	−9.68	−38.78 to 19.38		
Diuretic	0.97	−18.99 to 20.94		
Beta-blocker	16.26	−1.70 to 34.21		
Alpha-blocker	21.44	−13.64 to 56.51		
RAS inhibitor	21.67	3.82 to 39.51	14.08	−2.46 to 30.62
Lipid lowering therapy	0.95	−17.06 to 18.95		
Vitamin D administration	5.15	−14.16 to 22.45		
Phosphate binder	17.82	−0.420 to 36.05	16.87	0.14 to 33.56
Platelet aggregation inhibitor	10.35	−8.44 to 29.13		
Coumarine derivates	22.50	−14.09 to 59.08		
Iron supplements	22.56	3.81 to 41.32		
***Hemodynamic measurements***				
Systolic blood pressure (mm Hg)	0.54	0.08 to 1.00	0.37	−0.77 to 0.82

The B reflects the change of total LVM (in grams) related with one unit increment of the determinant.

R^2^ of the multivariable model = 0.22.

Table 4 shows that hsCRP, Il-6, hepcidin-25 and CTGF were not related to LVM.

**Table 4:** Hepcidin, hsCRP, Il-6 and CTGF as determinants of LVM.

	Univariable model		Adding to ‘basic’ multivariable model		
Determinant	B	95% CI	B	95% CI	ΔR^2^
Hepcidin-25 (nM)	−0.04	−0.46 to 0.38	0.04	−0.38 to 0.45	−0.003
hsCRP (mg/L)	0.22	−0.46 to 0.90	0.07	−0.43 to 0.57	−0.003
Il-6 (pg/mL)	0.03	−0.17 to 0.22	0.06	−0.13 to 0.23	−0.002
CTGF (nmol/L)	0.05	−3.92 to 4.01	0.67	−3.45 to 4.78	−0.001

The B reflects the change of total LVM (in grams) related with one unit increment of the determinant.

## Discussion

The present study confirmed the relation between a high LVM and outcome [2], [4], [31], [32]. Furthermore we expanded the evidence for a strongly increased risk of sudden death in patients with a high LVM. After confirming that LVM was a strong predictor of cardiovascular and overall mortality we wanted to study what factors determine the magnitude of LVM, and in particular if these determinants were potentially modifiable. In our analysis, factors related to LVM were: male gender, history of kidney transplantation, residual kidney function (RKF), albumin and use of phosphate binders. Thus we did not find determinants of LVM that could easily be altered in daily clinical practice. Lastly, we explored whether novel markers of inflammation, fibrosis and iron homeostasis (hsCRP, Il-6, CTGF and hepcidin-25), which in theory could lead to a higher LVM, were related to LVM in a large population of hemodialysis patients. Apparently, although hsCRP, Il-6, CTGF, hepcidin-25 have previously been found to be associated with cardiovascular damage, no relation exists between these biomarkers and the magnitude of LVM in ESKD patients.

### LVM and clinical events

A summary of previous papers in which the relation between left ventricular geometry and clinical events was studied in dialysis patients is shown in Table 5. Foley et al studied the relation between LVM and mortality risks in 433 ESKD patients and found a significant linear association between LVM and overall mortality as well as cardiovascular mortality in particular [2]. Zoccali et al studied the prognostic impact of LVM indexed for body surface are or height^2.7^ in 254 dialysis patients and found that both types of LVMi were related to both overall mortality and cardiovascular mortality [31].

**Table 5:** Summary of previous studies in which the relation between LV geometry and clinical events was examined in dialysis patients.

Author	patient nr	LV measurement	event	Risk measure	Conclusion
Silverberg et al	133	LVMi (g/m^2^)	mortality	RR: 2.9 (p = 0.013)	LVH is an important determinant of survival
1989 (33)			CV mortality	RR: 2.7 (0.08)	in incident dialysis patients
Foley et al	433	LVMi (g/m^2^)	mortality	RR: 1.003 (p = 0.11)	LVH is highly prevalent in th dialysis
1995 (2)			late (>2 yr) mortality	RR: 1.009 (p<0.001)	population and is a risk factor for mortality
London et al	153	more than 10% decrease	mortality	RR: 0.78 (p = 0.001)	partial regression of LVM has a favorable
2001 (4)		in LVMi (g/height^2.7^)	CV mortality	RR: 0.72 (p = 0.002)	effect on mortlity and CV-mortality
Zoccali et al	254	LVMi (g/m^2^)	mortality	HR: 1.01 (p<0.001)/1.03 (p<0.001)	LVM indexed for height^2.7^ provides a more
2001 (32)		LVMi (g/height^2.7^)	CV mortality	HR: 1.01 (p<0.001)/1.03 (p<0.001)	powerful predictor for death and CV events
			CV event	HR: 1.00 (ns)/1.02 (p = 0.004)	compared to LVM indexed for BSA
Zoccali et al	161	in top 75% progression	mortality	HR: 3.07 (p = 0.008)	Changes in LVMi have an independent
2004 (3)		in LVMi (g/height^2.7^)	CV event	HR: 3.02 (p = 0.02)	prognostic value for death and CV events

CV events are defined as a combination of both fatal and non-fatal cardiovascular events.

BSA: body surface area; CV: cardiovascular; HR: hazard ratio; LV: left ventricular; LVH: left ventricular hypertrophy; LVM: left ventricular mass; LVMi: left ventricular mass index; nr: number; RR: relative risk.

We are among the first to describe the relationship between LVM and sudden death specifically in ESKD patients. In fact, ESKD patients in the highest tertile of LVM had an almost 14-fold higher risk of sudden death when compared to the lowest LVM tertile, while their risk of dying from a cardiac cause in general was ‘only’ increased by a factor 3.5. The underlying mechanism may be through a decrease in myocardial capillary density, diastolic and systolic dysfunction, disturbances in interventricular conduction, chamber dilatation and eventually more compensatory hypertrophy. These processes lead to an increased risk of triggering a fatal arrhythmia [1], [33]. Autopsy studies in ESKD patients point to the presence of diffuse inter-myocardiocyte fibrosis specific for this group, which may indicate an electrical instability predisposing to sudden death [34]. The percentage of sudden deaths (56%) from all cardiac deaths in our population was similar to those of earlier studies [33].

For a combination of fatal- and non-fatal cardiovascular events no relation with LVM size was found. To our knowledge, no such relation has been described in earlier literature; although Zoccali et al found a significant relation between LVM indexed for height^2.7^ and fatal- and non-fatal cardiovascular events combined [31]. Since there were only 3 lethal CHD events in our study, this association could not be explored in our population.

### Determinants of LVM

Factors related to LVM were: male gender, history of kidney transplantation, residual kidney function (RKF), albumin and use of phosphate binders.

It was a surprising finding that a history of CVD and blood pressure (BP) were not found to be associated with LVM. Regarding the lack of relation between LVM and CVD this could be attributed to the fact that our definition of CVD encompassed several periphery vasculature diseases/interventions, which do not necessarily lead to an enlargement of LVM. Also, many ESKD patients have a high LVM without a history of CVD [2]. While BP is very variable over time in dialysis patients (mostly due to rigorous changes in extracellular volume during and in-between dialysis treatments), our BP results are an average of three pre- and three post-dialysis BP measurements. Hence our BP measurements could be a poor representative of the total BP burden of a patient (which is truly related to LVM).

The relation between LVM and a history of kidney transplantation [35], [36] and albumin [37] is in accordance with earlier literature.

The positive relation between LVM and RKF may be explained by a ‘survivor bias’: patients that still have RKF have been on dialysis for a shorter period of time. As time passes, the patients with a high LVM are more likely to die, the patient with a lower LVM remain and lose their RKF. In our population, the dialysis vintage differs significantly between patient with RKF (1.92±1.58 years) and without RKF (4.00±3.4 years).

Previous studies on predictors of LVM and LVMi in HD patients identified phosphate and the calcium-phosphate product as patient characteristics associated with LVH [38]–[40]. In our analysis however, these laboratory values were not significantly related to LVM, while there was a positive association between LVM and use of phosphate binders. The serum calcium and phosphate are well controlled in our dialysis population, and phosphate binders were prescribed to 74% of the patients (mainly sevelamer, a non-calcium containing phosphate binder: 54%). Hyperphosphatemia can lead to vascular calcification and myocardial fibrosis, resulting in increased cardiovascular risk [41]. Thus, it is plausible that in our population the prescription of phosphate binders is a reflection of higher phosphate intake at present and/or hyperphosphatemia in the past, resulting in higher LVM.

### Relation between LVM and hsCRP, Il-6, CTGF, hepcidin

We are among the first to investigate the association between LVM and the biomarkers hsCRP, Il-6, CTGF and hepcidin in a population of ESKD patients, which is also large enough to perform appropriate corrections for clinically relevant variables without creating an overfitted model. Although there is a theoretical incentive, as described in the Introduction, to hypothesize that these biomarkers may contribute to LVM, we do not find such a relation in our population. Apparently, although hsCRP, Il-6, CTGF, hepcidin-25 have previously been found to be associated with cardiovascular damage, no relation exists between these biomarkers and the magnitude of LVM in ESKD patients.

In earlier papers concerning LVM and prognosis, LVM was indexed for body surface are, or divided by height^2.7^. It was shown that these indexations, especially LVM/height^2.7^ are better predictors of clinical events than LVM. [3], [4]) A downside of ratios is that observed relation may be due to the nominator, the denominator or both. Therefore in the present analyses we chose to use LVM for our analyses only with correction for height and weight in the propensity scores for optimal statistical adjustment. As shown in Tables S1a and S1b in File S1, when LVM was adjusted for height and weight, the relation with clinical events was similar to that of LVM indexed for BSA or height^2.7^.

### Strengths and limitations

This study had several limitations. First, 7.7% of data was missing and biomarkers were measured in only 75.5% of the patients. However, since multiple imputation was performed for missing variables included in the multivariable analysis, this prevents the drawing of wrong conclusions due to the fact that data may be missing in specific patients for a reason, and not by chance and by increasing the power of our analyses [29]. Furthermore, our sensitivity analyses of complete cases showed no marked differences with the regression performed on the imputed data. Second, the number of CHD events and sudden deaths was small, thus limiting the precision of our estimates. Third, since cross-sectional data was used to determine variables related to LVM, causality of relations cannot be established. Fourth, measurements of LVM by echocardiography is less precise and reliable than measurement by cardiac magnetic resonance imaging (CMRI) [1]. However, while CMRI is recognized as the “gold standard” for ventricular geometry measurements, it is less often applied in clinical practice since it is more expensive, not widely available and has contra-indications such as claustrophobia and use of cardiac implantable devices [1]. Thus it was not feasible to perform CMRI measurements in our relatively large cohort of dialysis patients. This may have led to misclassification, which generally leads to an underestimation of the magnitude of the relations under study.

The strengths of this study are the large sample size, the concise and prospective data collection, the independent review of source documentation for all primary and secondary outcomes and the double independent analysis of the echocardiography recordings blinded for patient characteristics.

## Conclusion

In this study we confirmed the relation between LVM and all-cause mortality. Furthermore we demonstrated a markedly increased risk of sudden death in patients with a high LVM.

No relationship was found for markers of inflammation (except for a negative association with albumin) and fibrosis.

## Supporting Information

File S1 **Appendix S1, Names of the ethics committees/institutional review boards. Table S1, (A) Hazard ratio of clinical events by LVMi in grams per m^2^ divided into tertiles.** (B) Hazard ratio of clinical events by LVMi in grams per height^2.7^ divided into tertiles. **Table S2, Whole-case analysis (n = 289) of determinants of LVM in dialysis patients: univariable and multivariable regression analysis.**
(DOCX)
