# Supplementary material for: Left Ventricular Mass in Dialysis Patients, Determinants and Relation with Outcome. Results from the COnvective TRansport STudy (CONTRAST)
Source: PLoS One. 2014 Feb 5;9(2):e84587. doi: 10.1371/journal.pone.0084587 (PMC3914777; doi:10.1371/journal.pone.0084587)
Supplement: File S1 — Appendix S1, Names of the ethics committees/institutional review boards. Table S1, (A) Hazard ratio of clinical events by LVMi in grams per m2 divided into tertiles. (B) Hazard ratio of clinical events by LVMi in grams per height2.7 divided into tertiles. Table S2, Whole-case analysis (n = 289) of determinants of LVM in dialysis patients: univariable and multivariable regression analysis. (DOCX) [file pone.0084587.s001.docx]

**Supplement**

Appendix S1: Names of the ethics committees / institutional review boards.

This study is a substudy of CONTRAST, and was performed in 15 centers. The names of the ethics committees / institutional review boards are listed below:

-Medisch Ethische Toetsings Commissie Vrije Universiteit Medisch Centrum (the central medical ethics review board)

-Medisch Ethische Toetsingscommissie Medisch Centrum Rijnmond-Zuid Rotterdam (MCRZ)

-Medisch Ethische Commissie Academisch Medisch Centrum

-Beoordelingscommissie Wetenschappelijk Onderzoek (BCWO) Gelderse Vallei Ede

-Medisch Ethische Toetsingscommissie Ziekenhuis Leyenburg Den Haag

-Medisch Ethische Toetsingscommissie Martini Ziekenhuis Groningen

-Medisch Ethische Toetsingscommissie Noord-Holland Alkmaar (Medisch Centrum Alkmaar)

-Medisch-Ethische Toetsingscommissie Onze Lieve Vrouwe Gasthuis Amsterdam

-Commissie Mensgebonden Onderzoek Nijmegen (UMC St. Radboud)

-Medisch Ethische Toetsingscommissie UMC Utrecht

-Commissie Experimenteel Onderzoek op Mensen (CEM) Venlo (VieCuri Medisch Centrum voor Noord Limburg)

-Lokale Toetsingscommissie Alysis Zorggroep Arnhem (Ziekenhuis Rijnstate Arnhem)

-ICH/Good Clinical Practice Commissie Slingeland Ziekenhuis Doetinchem

-Medisch Ethische Commissie Rijnland Ziekenhuis Leiderdorp

-Comités d'evaluation scientifque et d'éthique de la recherche - Hôpital Notre-Dame du CHUM (Centre Hospitalier de l'université de Montréal) Montréal Canada

Table S1a: Hazard ratio of clinical events by LVMi in grams per m^2^ divided into tertiles.

|  | **T1: <108** | **T2: 108<LVMi<142** | **95% CI** | **T3: >142** | **95% CI** |
| --- | --- | --- | --- | --- | --- |
| **Crude** |  |  |  |  |  |
| Mortality | 1 | 1.38 | 0.89 - 2.18 | 2.16* | 1.40 - 3.34 |
| Cardiovascular death | 1 | 1.46 | 0.61 – 3.46 | 2.98* | 1.37 – 6.48 |
| Sudden death | 1 | 3.28* | 1.09 – 12.13 | 4.83* | 2.36 – 17.13 |
| Cardiovascular events | 1 | 1.06 | 0.67 – 1.70 | 1.51 | 0.98 - 2.35 |
| CHD events | 1 | 1.07 | 0.53 – 2.19 | 1.01 | 0.50 – 2.03 |
| **Adjusted^a^** |  |  |  |  |  |
| Mortality | 1 | 1.10 | 0.91 – 2.36 | 1.46* | 1.16 - 2.59 |
| Cardiovascular death | 1 | 1.24 | 0.48 – 3.13 | 2.62* | 1.10 – 6.24 |
| Sudden death | 1 | 2.42 | 0.83 – 9.27 | 3.30* | 1.09 – 12.33 |
| Cardiovascular events | 1 | 1.00 | 0.60 – 1.07 | 1.35 | 0.82 - 2.22 |
| CHD events | 1 | 0.96 | 0.44 – 2.11 | 0.79 | 0.35 – 1.78 |

*p<0.05

^a^Adjusted with a propensity score containing determinants of LVMi (male gender, residual renal function, history of kidney transplantation, albumin, use of RAS-inhibitors, use of phosphate binders, systolic blood pressure) and history of cardiovascular disease, diabetes and dialysis modality (intervention).

Table S1b: Hazard ratio of clinical events by LVMi in grams per height^2.7^ divided into tertiles.

|  | **T1: <48** | **T2: 48<LVMi<64** | **95% CI** | **T3: >64** | **95% CI** |
| --- | --- | --- | --- | --- | --- |
| **Crude** |  |  |  |  |  |
| Mortality | 1 | 1.28 | 0.81 – 2.01 | 1.90* | 1.23 – 2.93 |
| Cardiovascular death | 1 | 2.12 | 0.91 – 4.95 | 2.71* | 1.18 – 6.19 |
| Sudden death | 1 | 3.54* | 1.07 – 12.86 | 4.17* | 1.17 – 14.96 |
| Cardiovascular events | 1 | 1.11 | 0.70 – 0.75 | 1.30 | 0.83 – 2.03 |
| CHD events | 1 | 1.26 | 0.62 – 2.35 | 0.90 | 0.45 – 1.80 |
| **Adjusted^a^** |  |  |  |  |  |
| Mortality | 1 | 1.04 | 0.64 – 1.69 | 1.33* | 1.08 – 2.31 |
| Cardiovascular death | 1 | 2.08 | 0.84 – 5.16 | 2.93* | 1.09 – 7.24 |
| Sudden death | 1 | 2.64 | 0.65 – 9.32 | 2.94* | 1.79 – 10.96 |
| Cardiovascular events | 1 | 1.13 | 0.69 – 1.86 | 1.06 | 0.64 – 1.75 |
| CHD events | 1 | 0.91 | 0.39 – 2.14 | 0.76 | 0.35 – 1.64 |

*p<0.05

^a^Adjusted with a propensity score containing determinants of LVMi (male gender, residual renal function, history of kidney transplantation, albumin, use of RAS-inhibitors, use of phosphate binders, systolic blood pressure) and history of cardiovascular disease, diabetes, post-dialysis weight and dialysis modality (intervention).

Table S2: Whole-case analysis (n=289) of determinants of LVM in dialysis patients: univariable and multivariable regression analysis. The B reflects the change of total LVM (in grams) related with one unit increment of the determinant.

|  | **Univariable model** | | **Multivariable model** | |
| --- | --- | --- | --- | --- |
| **Determinant** | **B** | **95% CI** | **B** | **95% CI** |
| ***Demographic data*** |  |  |  |  |
| Male gender (n=327) | 56.47 | 39.03 to 73.90 | 54.17 | 37.30 t0 71.03 |
| Race, caucasian (n=327) | 12.92 | -9.75 to 35.60 |  |  |
| Age, years (n=327) | 0.75 | 0.08 to 1.42 |  |  |
| Smoking (n=311) | 24.51 | -1.85 to 47.18 |  |  |
| ***Dialysis Properties*** |  |  |  |  |
| Duration of dialysis, hours (n=321) | 36.84 | 13.02 to 60.66 |  |  |
| spKt/Vurea (n=324) | -102.8 | --146.1 to -59.48 |  |  |
| AV fistula (n=327) | 17.59 | -4.66 to 38.83 |  |  |
| ***Comorbidities*** |  |  |  |  |
| Cardiovascular disease (n=327) | 16.54 | -1.50 to 34.58 |  |  |
| Diabetes (n=314) | 2.65 | -18.36 to 23.66 |  |  |
| Previous kidney transplant (n=327) | -49.76 | -80.38 to -19.01 | -42.96 | -72.35 to -13.55 |
| Dialysis vintage, years (n=326) | -5.45 | -8.61 to -2.30 |  |  |
| Residual kidney function (n=326) | 29.28 | -11.52 to 47.04 | 19.26 | 2.29 to 36.23 |
| ***Laboratory parameters*** |  |  |  |  |
| Hemoglobin, g/dL (n=326) | -1.00 | -12.58 to 10.58 |  |  |
| Phosphate, mmol/L (n=326) | 0.88 | -17.16 to 18.93 |  |  |
| Calcium, mmol/L (n=327) | 13.37 | -32.36 to 63.99 |  |  |
| Calcium*Phosphate (n=326) | 1.28 | -6.49 to 9.03 |  |  |
| Albumin, g/L (n=325) | -1.99 | -4.22 to 0.24 | -2.80 | -4.86 to -7.41 |
| Creatinin, μmol/L (n=325) | -0.02 | -0.05 to 0.02 |  |  |
| ***Therapeutic parameters*** |  |  |  |  |
| Erythropietin (n=325) | -9.68 | -38.84 to 19.49 |  |  |
| Diuretic (n=325) | 0.97 | -18.98 to 20.94 |  |  |
| Beta-blocker (n=325) | 16.26 | -1.70 to 34.23 |  |  |
| Alpha-blocker (n=325) | 21.15 | -13.77 to 56.64 |  |  |
| RAS inhibitor (n=325) | 21.67 | 3.75 to 39.58 | 12.09 | -5.40 to 29.58 |
| Lipid lowering therapy (n=325) | 1.66 | -16.37 to 19.90 |  |  |
| Vitamin D administration (n=325) | 5.15 | -14.29 to 24.52 |  |  |
| Phosphate binder (n=325) | 17.82 | -0.50 to 36.12 | 19.83 | 3.09 to 36.66 |
| Platelet aggregation inhibitor (n=325) | 10.35 | -8.41 to 29.18 |  |  |
| Coumarine derivates (n=325) | 22.50 | -14.22 to 59.22 |  |  |
| Iron supplements (n=325) | 22.56 | 3.74 to 41.39 |  |  |
| ***Hemodynamic measurements*** |  |  |  |  |
| Systolic blood pressure, mmHg (n=327) | 0.54 | 0.08 to 1.00 | 0.32 | -0.13 to 0.77 |

R² of the multivariable model = 0.19.

For univariate analyses, all the cases for which the variable was complete were included in the analysis.
